# Plant-Derived *Lactobacillus paracasei* IJH-SONE68 Improves the Gut Microbiota Associated with Hepatic Disorders: A Randomized, Double-Blind, and Placebo-Controlled Clinical Trial

**DOI:** 10.3390/nu14214492

**Published:** 2022-10-26

**Authors:** Narandalai Danshiitsoodol, Masafumi Noda, Keishi Kanno, Tomoyuki Uchida, Masanori Sugiyama

**Affiliations:** 1 Department of Probiotic Science for Preventive Medicine, Graduate School of Biomedical and Health Sciences, Hiroshima University, Kasumi 1-2-3, Minami-ku, Hiroshima 734-8551, Japan; 2 Department of General Internal Medicine, Hiroshima University Hospital, Kasumi 1-2-3, Minami-Ku, Hiroshima 734-8551, Japan; 3 Department of Clinical Pharmaceutical and Therapeutics, Hiroshima University, Kasumi 1-2-3, Minami-ku, Hiroshima 734-8551, Japan

**Keywords:** *Lactobacillus paracasei*, gut microbiota, clinical trial, plant-derived lactic acid bacterium, hepatic disorders, *Anaerostipes*, *Veillonella*

## Abstract

Our previous clinical study has shown that the exopolysaccharide (EPS) produced by a plant-derived lactic acid bacterium, *Lactobacillus paracasei* IJH-SONE68, improves chronic allergy status in humans. In addition, an inhibition of visceral fat accumulation was observed following the intake of EPS during animal experimentation. In the present study, we have further evaluated the health-promoting effects of a spray-dried powder of pineapple juice that is fermented with the IJH-SONE68 strain. This was conducted in a double-blind, randomized, placebo-controlled, parallel-group clinical trial at Hiroshima University from May 2019 to July 2021. Eighty healthy volunteers at range of ages 23–70, with a body mass index between 25 and 29.99, were enrolled. After the 12 weeks of the experimental period were complete, although the average visceral fat area in both groups similarly decreased, there was no significant difference in the content of visceral fat area or in the obesity-related physical parameters in both groups. Further, we found that the serum liver function indices (AST and ALT) in the test group decreased within a statistically determined trend (*p* = 0.054). The fecal microflora analysis revealed, in the test group, a statistically significant increase in the relative abundance changes within *Anaerostipes*, which has been reported to help suppress hepatic inflammation.

## 1. Introduction

Lactic acid bacteria (LABs), which are generally non-pathogenic and Gram positive, are known to be useful for human health [1,2,3] and are recognized as a probiotic [4]. Depending on its abundance in various habitats, LAB strains possess different functional properties in host organisms. LAB of human and animal origin have been reported as able to assimilate lactose and are thus broadly used in dairy product manufacturing [5,6]. On the other hand, LAB reside in low numbers within the surface of the plants. It has been demonstrated that LABs from plant origin are able to grow in low temperatures and have gene sets for the degradation of complex plant polymers. Moreover, the defense and stress response genes are significantly different from the genes in animal origin variants with respect to their adaptation on substrates [7].

We isolated more than 1200 LAB strains from plant sources, such as fruits, vegetables, grains, and medicinal plants and subsequently established a plant-derived LAB library. The LAB strains that were isolated can grow in vegetable and fruit juices as a culture medium. Further, our previous studies demonstrated, through randomized clinical trials, the health-beneficial effects of several plant-derived LAB isolates [8,9,10]. One of the isolates, the fig leaf-derived *Lactobacillus* (*L.*) *paracasei* IJH-SONE68, was found to produce unique structural exopolysaccharide (EPS) outside cells [11]. We have also shown that the intake of IJH-SONE68-derived EPS prevents and ameliorates inflammatory responses in both picryl chloride-induced contact dermatitis and dextran sulfate sodium (DSS)-induced ulcerative colitis in model mice. This was achieved via the reduction in the accelerated expression of inflammatory cytokines [12,13]. Our clinical study involving volunteer participants with perennial allergy symptoms also showed that the EPS from the IJH-SONE68 strain improved allergic conditions [14].

Obesity is a major public health problem caused by both hereditary and environmental factors. Recent studies conducted in human and in vivo clinical trials have revealed the anti-obesity effects of dietary supplementation with probiotics [15,16,17]. In our previous study, the oral administration of pineapple juice fermented with the IJH-SONE68 strain significantly reduced weight gain and visceral fat accumulation in high-fat diet-induced obesity model mice (see Supplementary Data). Further, a randomized, double-blind, placebo-controlled study conducted by Mo et al. demonstrated that the intake of a high dose of kimchi-isolated LAB strains resulted in the reduction in several obesity parameters when compared with the placebo group. Further, their study also showed the modulation of the gut microbiota characteristics [18].

Judging from the clinical studies, the IJH-SONE68-derived EPS can support the health care of persons with not only perennial allergies, but also other chronic inflammation. On the other hand, since obesity has been regarded as a disease associated with different degrees of low-grade chronic inflammation [19,20], an aim of the present study is to show, therefore, whether the IJH-SONE68-derived EPS can improve obesity indices, anti-inflammatory effects, and other obesity-related factors by conducting a clinical study with overweight participants.

## 2. Materials and Methods

### 2.1. Participants

Healthy volunteers, between 23–70 years old, were recruited in the city of Hiroshima, Japan, through a series of advertisements. The inclusion criteria for participants were that they were healthy males or females with a body mass index (BMI) between 25 and 29.99 kg/m^2^. The exclusion criteria were as follows, participants who: (1) have allergic hypersensitivity to pineapple juice; (2) received management or treatment of hypertension, diabetes, or hyperlipemia by doctors; (3) take regular medication; (4) are pregnant or breastfeeding; (5) use medicines, supplements, or functional foods that may affect obesity indices and other obesity-related factors; or (6) took part in other clinical trials within 3 months of the commencement of this study. Written informed consent was obtained from all participants for the use of their clinical data in research.

### 2.2. Samples and Placebo

The test samples used in this clinical study were capsules containing a mixed powder of dextrin, the heat-killed IJH-SONE68 strain, and its cultured broth. The placebo capsules contained only dextrin. Both test and placebo capsules were produced by and purchased from the SAKURAO Brewery and Distillery Co., Ltd., Hiroshima, Japan. Pineapple juice was used for the IJH-SONE68 cultivation in sample preparation; then, the cultured broth was subjected to a spray dryer after heat treatment. A test diet capsule contained approximately 1.3 × 10^5^ cells of IJH-SONE68-containing powder (260 mg). The same amount of dextrin was used to fill the placebo capsules.

### 2.3. Study Design

This clinical trial was conducted as a double-blind, randomized, placebo-controlled, parallel-group study at Hiroshima University, Hiroshima, Japan, from May 2019 to July 2021. Participants who were eligible to be enrolled in this study based on the criteria were stratified based on BMI (27.5 ≤ or less) and then assigned to a test or placebo group using the blocked randomization method. The random allocation was generated by Microsoft Excel software in a 1:1 allocation ratio with a block size of 4. Only non-clinical staff without analytical involvement in this study carried out the randomization assignments; therefore, the participants and analytical staff were blinded to the assignments.

During the study period, participants were instructed to maintain their normal life and eating habits as much as possible and directed not to donate blood. They were also asked to consume their daily intake of four capsules containing a test sample or placebo at any time within the day for 12 weeks. Dated daily record forms were provided to participants to document the contents of their meals (including snacks and alcoholic drinks) for 3 days before the examination, their intake of capsules, and conditions throughout the study period.

A change in the visceral fat area was set as the primary outcome parameter. The secondary outcomes were changes in BMI, body fat percentage, waist circumference, blood glucose, serum lipids (triglyceride, total cholesterol, high-density lipoprotein (HDL) cholesterol, and low-density lipoprotein (LDL) cholesterol), serum liver function indices (aspartate aminotransferase (AST), alanine aminotransferase (ALT), and γ-glutamyl transpeptidase (γ-GTP)), and fecal microbiota. The participants were directed to visit Hiroshima University for physical examinations, biochemical measurements, and urinalysis every 4 weeks. Blood and urine samples were obtained after at least 9 h of fasting, and sent to SRL, Inc. (Tokyo, Japan) for clinical biochemical measurements and urinalysis using standard clinical methods. Body fat percentage and visceral fat area were measured using a body composition analyzer (BC-118E, Tanita, Tokyo, Japan) and visceral fat meter (EW-FA90, Panasonic, Osaka, Japan), respectively. The BC-118E analyzer measures body weight and bioelectrical impedance at first and then calculates body fat percentage via the multiple regression analysis, which is based on the correlation between the data obtained from the bioelectrical impedance analysis (BIA) and dual energy X-ray absorptiometry (DXA) methods [21]. The EW-FA90 meter calculates visceral fat area via a regression curve between measured abdominal bioelectrical impedance and estimated visceral fat area. Blood pressure was measured according to the Japanese Society of Hypertension Guidelines for the Management of Hypertension 2019 (JSH2019) [22], using a fully automatic sphygmomanometer (HBP-9020, Omron, Kyoto, Japan).

The protocol for this clinical study was approved by the Ethics Committee of Hiroshima University (approval no. C-267; date of approval: 22 March 2019) prior to advertisement. This study was registered in the University Hospital Medical Information Network Clinical Trials Registry (UMIN-CTR), ID: UMIN000036318 (date of registration: 11 March 2019) and was performed according to the guidelines of the Helsinki Declaration. According to the Common Terminology Criteria for Adverse Events version 5.0 (CTCAE v5.0), newly emerged or worsened adverse events after intervention were evaluated when those grades shifted higher.

### 2.4. Analysis of Fecal Microbiota Based on 16S rRNA Encoding Gene

Feces were collected at weeks 0 and 12 (before and after the intake period, respectively) within 3 days of the clinical visit. The 16S rRNA-based microbiota analysis was performed at Bioengineering Lab. Co., Ltd. (Kanagawa, Japan) using the Illumina Miseq sequence platform and Miseq Reagent Kit v3 (Illumina Inc., San Diego, CA, USA) with a 300 bp read length paired-end protocol. Briefly, the total DNA was extracted and purified from 200–500 mg of the fecal samples using a MPure-12 Automated Nucleic Acid Purification System and an MPure Bacterial DNA Extraction Kit (MP Biomedicals, Santa Ana, CA, USA). The V3–V4 region of 16S rRNA encoding genes was amplified using ExTaq HS (Takara Bio, Inc., Shiga, Japan) with the primers V3V4f_MIX (5′- ACACTCTTTCCCTACACGACGCTCTTCCGATCT -NNNNN- CCTACGGGNGGCWGCAG-3′) and V3V4r_MIX (5′- GTGACTGGAGTTCAGACGTGTGCTCTTCCGATCT -NNNNN- GACTACHVGGGTATCTAATCC-3’) under the following conditions (the underlined regions in each primer consist of random sequences with random bases): 2 min at 94 °C followed by 20–25 cycles of 30 sec at 94 °C, 30 sec at 55 °C, and 30 sec at 72 °C, and finally a 5 min extension period at 72 °C. The amplified DNA fragments were purified using an Agencourt AMPure XP (Beckman Coulter Inc., Brea, CA, USA) according to the manufacturer’s instructions. A 2 μL portion of the resultant purified fragments was further used as a template for the second PCR reaction using the primer set 5′-AATGATACGGCGACCACCGAGATCTACACXXXXXXXXACACTCTTTCCCTACACGACGC-3′ and 5′-CAAGCAGAAGACGGCATACGAGATXXXXXXXXGTGACTGGAGTTCAGACGTGTG-3′ under the following conditions (the underlined regions in each primer are index sequences designed to identify each sample in the analysis system): 2 min at 94 °C followed by 20–25 cycles of 30 sec at 94 °C, 30 sec at 55 °C, and 30 sec at 72 °C, and finally a 5 min extension period at 72 °C. The amplified samples were also purified and provided to the analytical instrument for analysis. After the analysis, taxonomic assignments were performed using the Quantitative Insights into Microbial Ecology (QIIME) 2.0 pipeline [23].

### 2.5. Procedures for Statistical Analysis

Prior to this study, the sample size was set at 48 per group in order to obtain 80% power in detecting a 15% difference in the visceral fat area. Further, the study would have a supposed standard deviation (S.D.) of 30% among the test group, which would mean a 95% confidence (two-sided) rate in using a two-sample *t*-test. The supposed difference and S.D. for sample size calculation were estimated using the data from our preliminary animal experiment (which is currently unpublished data). Except for fecal microbiota, the multiple-imputation method (with 20 data sets) was applied to missing data when the obtained data were analyzed in accordance with the intention-to-treat principle [24]; further, the resultant analyses were combined. The baseline characteristics were compared using Welch’s *t*-test [25], and changes from the baseline in each outcome were examined via analysis of covariance (ANCOVA) using each baseline value as a covariate. The changes in the relative abundance of each item analyzed in fecal microbiota were analyzed using the Mann–Whitney U test and Wilcoxon’s signed rank test for inter- and intra-group analyses, respectively. Fisher’s exact test was used for adverse events to assess differences between the two groups. All statistical analyses were performed using IBM SPSS Statistics 17.0 for Windows (IBM Japan, Tokyo, Japan).

## 3. Results

### 3.1. Recruitment and Baseline Characteristics of the Participants

**Figure 1:**
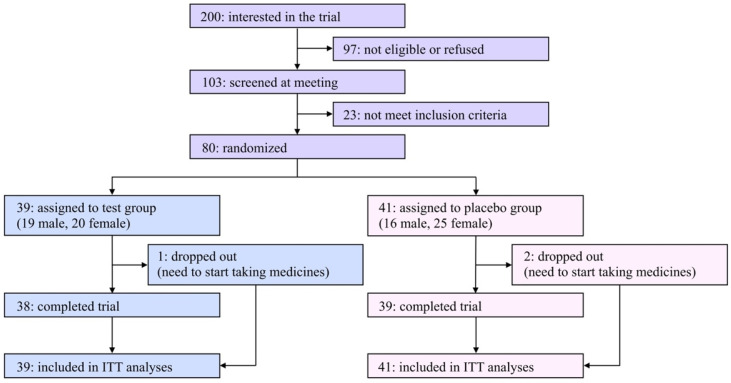
Flow diagram of participants in the present study.

A flow diagram of the participants of this study is shown in Figure 1. Two hundred applicants expressed interest in participating in this study. Among them, 80 eligible participants ages 23–70 were registered in this study after an explanatory meeting and screening and were randomly assigned to the test or the placebo group. Due to limited financial resources and a scheduled deadline, it was hard to recruit a satisfactory number of participants in the COVID-19 pandemic situation; therefore, the original registration goal (*n* = 100) was unavoidably abandoned.

The characteristics of the eligible participants at baseline are summarized in Table 1. Among the listed items, there were no significant differences between the two groups. During the trial period, three participants (one and two in the test and placebo groups, respectively) dropped out of the study due to the need to begin taking medicines, and the remaining 77 participants (96.3% from the baseline) completed the study. The rates of compliance in taking daily capsules were 97.8% and 95.4% in the test and placebo groups, respectively. The questionnaire asking which group each participant was assigned to resulted in 30.0% correct, 36.3% incorrect, and 33.8% “cannot be determined” answers, indicating that the blinding was successfully achieved.

### 3.2. Effect on Primary and Secondary Outcomes

Changes in the visceral fat area, which had been set as the primary outcome, during the 12 week trial period were compared between the two groups (Table 2). The average visceral fat area in both groups similarly decreased, and there was no significant difference in the changes (*p* = 0.830).

Changes in secondary outcomes are also summarized in Table 2. There were no significant changes or differences between the two groups in obesity-related physical measurements (BMI, body fat percentage, and waist circumference). Regarding the serum lipids and blood glucose, no notable changes were observed except for serum triglyceride, which increased in the placebo group but decreased in the test group. Serum liver function indices in the placebo group remained constant or increased slightly, whereas those in the test group were uniformly reduced; however, there were no significant differences between the two groups for changes in AST and ALT (*p* = 0.054).

### 3.3. Adverse-Effect Monitoring

To detect the adverse effects for the intake of the test and placebo capsules, blood pressure and other serum parameters were measured, and a urine dipstick test was also carried out. In accordance with CTCAE v5.0, there was no significant difference in adverse events having a possible relation to the study design or treatments between the two groups (Table 3). Although there were no abnormal changes in those monitored parameters throughout this study, notably, the average serum lactate dehydrogenase level was reduced in the test group and the change from the baseline is significantly different from that of the placebo group (see Supplementary Table S1).

### 3.4. Changes in Fecal Microbiota

The obtained sequences were analyzed and annotated as 14, 23, 31, 51, and 100 kinds of phylum, class, order, family, and genus, respectively. Among those 219 items, in terms of changes in relative abundance from the baseline, predominant differences were observed in 12 items (Epsilonproteobacteria at class; Campylobacterales at order; Odoribacteraceae, Campylobacteraceae, and Tissierellaceae at family; and *Veillonella*, *Anaerostipes*, *Dorea*, *Butyricicoccus*, *Lachnobacterium*, *Campylobacter*, and *Oxalobacter* at genus) between the placebo and test groups. However, the detection frequencies were quite low (only in 4–10 participants) in Epsilonproteobacteria, Campylobacterales, Campylobacteraceae, Tissierellaceae, *Lachnobacterium*, *Campylobacter*, and *Oxalobacter*; therefore, those taxa were omitted from the discussion.

**Figure 2:**
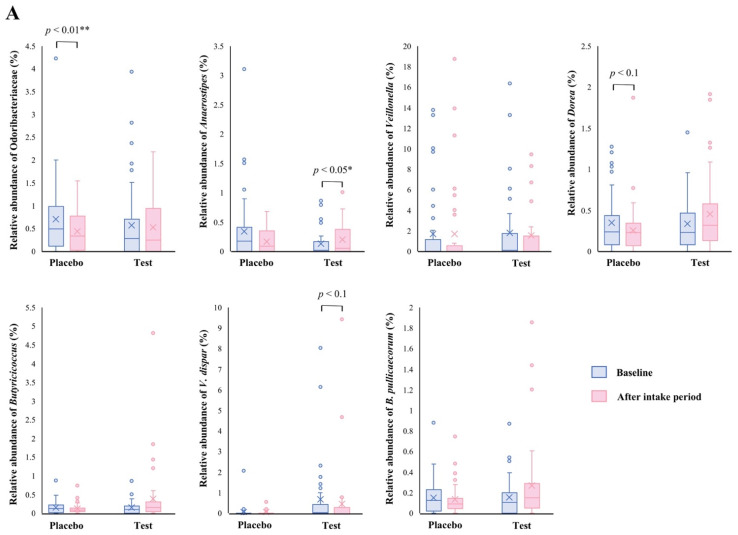

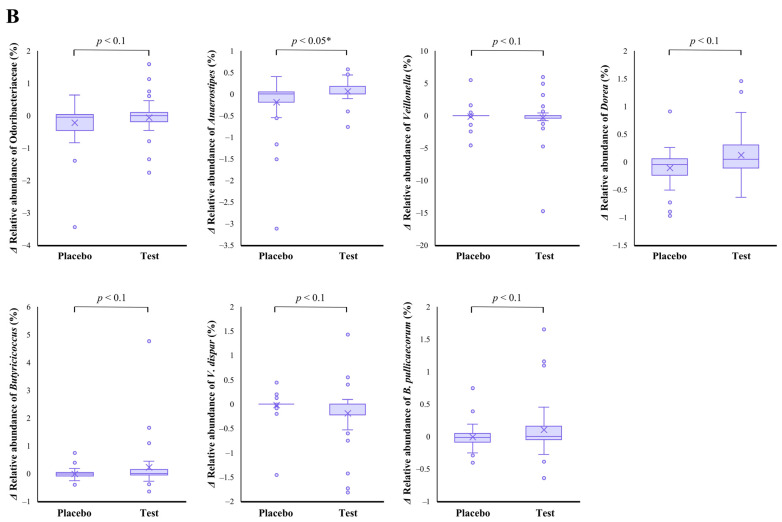
Comparison of relative abundance before and after the intake period (**A**) and the changes in relative abundance from the baseline of fecal microbiota (**B**) between the test (*n* = 39) and placebo (*n* = 41) groups on Odoribacteraceae, *Anaerostipes*, *Veillonella*, *Dorea*, *Butyricicoccus*, *Veillonella dispar*, and *Butyricicoccus pullicaecorum*. The statistical analyses were performed using the Mann–Whitney U test and Wilcoxon’s signed rank test for inter- and intra-group analyses, respectively (* *p* < 0.05 and ** *p* < 0.01). Data are shown as a box plot with medians (line inside boxes), means (cross mark), 25–75th percentile (limits of boxes), whiskers indicating a 95% data range, and outliers indicated by circles.

The family level analyses on the relative abundance before and after the intake period revealed that a significant decrease (*p* < 0.01 vs. the baseline) was observed in the placebo group in Odoribacteraceae (Figure 2A). The average abundance of Odoribacteraceae increased, relatively, in the test group compared with the placebo group after the intake period; however, the difference in changes was not significant (Figure 2B, *p* < 0.1).

At the genus level, statistical significance was observed only in *Anaerostipes*, specifically in regard to the difference in relative abundance changes. In this genus, a significant increase (*p* < 0.05 vs. the baseline) in the relative abundance during the intake period was observed in the test group (Figure 2A), and a significant difference in its changes between the two groups were also observed (Figure 2B, *p* < 0.05). In the *Dorea* and *Butyricicoccus* genera, based on the inter- and intra-group analyses, their abundance relatively increased in the test group through the trial, but no statistical differences were observed. Although among the listed genera, only the *Veillonella* genus displayed a slightly decreasing change in the test group when compared with that of the placebo group (Figure 2B. *p* < 0.1); further, there were no notable differences in the intra-group analysis before and after the study period in both groups (Figure 2A). However, a remarkable, but no significant, decrease was observed in *Veillonella dispar*—which is the only annotatable species in the genus *Veillonella* in the present study—in the intra- and inter-group analyses (Figure 2A, *p* < 0.1 vs. the baseline in the test group and Figure 2B, *p* < 0.1, respectively). On the other hand, although the analysis of the only annotatable species in the genus *Butyricicoccus*, *Butyricicoccus pullicaecorum* showed an inter-group difference between the test and placebo groups (Figure 2B, *p* < 0.1), no statistical difference was also observed in the intra-group analysis vs. the baseline (Figure 2A).

## 4. Discussion

Different from our recent preliminary study in high-fat diet-induced obesity mice (see Supplementary Data), significant inhibitions of weight gain and visceral fat accumulation were not observed in the present clinical study on IJH-SONE68-fermented pineapple juice. Due to the ease of handling and stable supply, the spray-dried powder was adopted as the sample form. The daily dose of fermented juice fed in the animal study was 200 μL/day/mouse, and the dose was equivalent to the human dose of 33 mL/day estimated by a human equivalent dose (HED) calculation [26]. The daily intake amount of four test capsules containing 1040 mg of IJH-SONE68-derived powder is equivalent to 2 mL of IJH-SONE68-fermented pineapple juice. Therefore, the dose in the present study is less than 1/10 of the effective dose in the animal study, which may be why no notable improvement was observed in most primary and secondary outcomes. In particular, the average visceral fat area and waist circumference in both groups similarly decreased. This is most likely due to the placebo effect that may raise participants’ awareness of losing weight, which is caused by participation in the present trial. In addition, the estimated average daily calorie intake of both groups also decreased without statistical significance (from 2026 to 1990 kcal/day in the test, and from 1970 to 1956 kcal/day in the placebo groups), thus the result also supports the possibility of psychological influence. However, not all of the outcome parameters were unaffected. As shown in Table 2 and Table 3, a serum triglyceride decrease was observed in the test group, and the number of participants who showed hypertriglyceridemia (grades 1 and 2) in the placebo group was higher than that in the test group (but without significance, *p* = 0.080).

Interestingly, although biochemical measurements were neither primary nor secondary outcomes, and were monitored only to detect adverse effects, the recent study using the same sample and placebo [14] also revealed that serum levels of AST and ALT were significantly decreased by IJH-SONE68 intake (*p* = 0.001 and 0.022, respectively). Considering the previous and present results together, IJH-SONE68-derived powder seems to have beneficial effects on both parameters. The fecal microbiota analysis performed in the present study provides helpful information to better characterize its health-promoting effect, as follows.

Our results showed that the relative abundance of the *Anaerostipes* genus was significantly increased in the test group (Figure 2). The *Anaerostipes* genus consists of a butyrogenic, Gram-positive, obligate anaerobe and is one of the major 15 abundant taxa in a healthy human microbiome [27]. The *Anaerostipes* species can convert polymeric sugars, such as inulin, into not only lactate and acetate, but also short-chain fatty acids (SCFAs), such as propionate and butyrate [28,29,30]. The SCFAs can potentially contribute to maintaining healthy intestinal permeability via suppressing mucosal inflammation [31]. Zhang et al. have reported the improving effects of shenling baizhu powder, which is a traditional Chinese medicine, on liver function and inflammation in high-fat diet-induced non-alcoholic fatty liver disease (NAFLD) model mice, specifically through the increased abundance of fecal *Anaerostipes* and *Bifidobacterium* genera [32]. In addition, *Bifidobacterium* and *Anaerostipes* species produce SCFAs; therefore, increasing those bacteria is suggested as a possibility in which to help suppress hepatic inflammation.

Clinical microbiota profiling also revealed the relationship between hepatic diseases and the relative abundance of *Anaerostipes* [33,34]. When compared with patients during the progression of chronic hepatitis B disease, healthy individuals had a higher distribution of *Anaerostipes* in their fecal microbiota [33]. Moreover, the abundance of stool *Anaerostipes* was not correlated with AST and ALT levels (*p* < 0.05) in primary biliary cholangitis (PBC) patients [34]. It is of more interest that the relative abundance of the *Anaerostipes* species has reportedly decreased in infants and children suffering from food or respiratory allergies and eczema [35,36,37,38,39]. Although a gut microbiota analysis was not performed in our recent clinical study on allergic symptoms [14], it is expected that similar changes may also have occurred in the subjects, suggesting that an increase in *Anaerostipes* contributed to the improvement in hepatic parameters and allergic status.

The relative abundance of *V. dispar*, which is an obligate, anaerobic, Gram-negative coccus, declined, relatively, in the test group during the study period (Figure 2). The bacterium is involved in only a few diseases [40], whereas many correlations between its abundance in the gut and hepatic disorders—such as hepatic B liver cirrhosis [41], primary sclerosing cholangitis [42], and primary biliary cholangitis [43]—have been reported. In particular, in autoimmune hepatitis, *V. dispar* has been shown to be the most significant species that is associated with the severity of the disease and to have a positive correlation with the serum AST level [44]. These observations are suggested to be as a result of the increased intestinal permeability, and subsequent bacterial translocation, caused by increased lipopolysaccharide (LPS) production and the dysfunction of tryptophan and arginine metabolisms [44,45]. The positive correlation between the abundance of *Veillonella* and increased systemic inflammation, endotoxemia, and hepatic encephalopathy has become clear [46,47,48]; therefore, the *Veillonella* genus appears to contribute to the development of not only hepatitis but also other inflammatory disorders. The observed decline in *V. dispar* in the present study was not much, however it is expected to be involved in relieving inflammation.

When compared with the period before the trial period began, changes in the abundance of Odoribacteraceae and *Dorea* decreased with (*p* < 0.01) and without (*p* < 0.1) statistical significance, respectively, in the placebo group. The abundance of two *Dorea* species, *Dorea longicatena* and *Dorea formicigenerans*, has been reported to be negatively correlated with hepatic parameters in metabolic dysfunction-associated fatty liver diseased (MAFLD) patients [49]. On the other hand, Ahn et al. have reported that different species of *Dorea* are associated with different effects on obesity-related and hepatic conditions in NAFLD patients [50], suggesting that a wide variety of effects associated with gut microbiota depend not on the bacterial genus but on the species, at least in the *Dorea* genus. It has been reported that the family Odoribacteraceae can produce a potent antimicrobial bile acid and isoallolithocholic acid [51]. Additionally, its higher abundance is specific to long-lived elderly people [52]. This family was newly established by Munoz et al. in 2016 [53]; thus, further characteristics and the health-promoting effects of the family will be reported in further studies.

Statistically significant improvements in the visceral fat area as the primary outcome, and other obesity-related indices, were not found in the present trial. However, intake of the spray-dried powder prepared from the fermented broth of the IJH-SONE68 strain has been suggested to alter the gut microbiota to, therefore, improve hepatic inflammation. Regarding the hypothetical anti-inflammatory effect on the liver, the changes in fecal microbiota observed in the present study and the improving effect of the IJH-SONE68-derived EPS against contact dermatitis [12] and ulcerative colitis [13] may properly support the hypothesis. As the improving effect of the IJH-SONE68-derived EPS in hepatic parameters was unexpected before the present trial (including preliminary animal experiments), we are thus going to further perform the clinical trial with people who have relatively high AST and ALT in order to confirm whether EPS surely improves the hepatic parameters.

## Figures and Tables

**Table 1:** The characteristics of the eligible participants at baseline.

	Test (*n* = 39)	Placebo (*n* = 41)	*p* Value
Age (y)	55.8 ± 10.2	55.3 ± 12.1	0.840
Male	56.3 ± 17.8 (*n* = 19)	54.1 ± 13.8 (*n* = 16)	0.621
Female	55.4 ± 8.6 (*n* = 20)	56.1 ± 11.1 (*n* = 25)	0.808
Height (cm)	163.7 ± 8.6	159.9 ± 9.3	0.060
Body weight (kg)	72.3 ± 9.7	68.8 ± 8.1	0.084
BMI (kg/m^2^)	26.9 ± 1.4	26.9 ± 1.5	0.985
Body fat percentage (%)	31.8 ± 6.8	33.4 ± 7.1	0.317
Waist circumference (cm)	94.5 ± 5.8	94.7 ± 5.2	0.900
Visceral fat area (cm^2^)	134.6 ± 41.0	132.4 ± 13.9	0.793
Systolic blood pressure (mmHg)	125.0 ± 15.2	126.0 ± 13.9	0.765
Diastolic blood pressure (mmHg)	76.8 ± 12.2	76.3 ± 10.3	0.851

Data are indicated as mean ± S.D. *p* values are calculated by the Welch’s *t*-test.

**Table 2:** Changes in primary and secondary outcomes.

	Test (*n* = 39)	Placebo (*n* = 41)	*p* Value
Visceral fat area (cm^2^)			0.830
Baseline	134.6 ± 6.6	132.4 ± 5.3	
Change at 12 week	−3.7 ± 2.6	−4.5 ± 2.5	
BMI (kg/m^2^)			0.958
Baseline	26.86 ± 0.23	26.87 ± 0.24	
Change at 12 week	−0.087 ± 0.09	0.080 ± 0.09	
Body fat percentage (%)			0.389
Baseline	31.9 ± 1.1	33.4 ± 1.1	
Change at 12 week	0.04 ± 0.22	0.31 ± 0.22	
Waist circumference (cm)			0.222
Baseline	94.5 ± 0.9	94.7 ± 0.8	
Change at 12 week	−0.61 ± 0.47	−1.40 ± 0.45	
Blood glucose (mg/dL)			0.809
Baseline	105.2 ± 1.6	102.0 ± 1.1	
Change at 12 week	−0.42 ± 0.93	−0.74 ± 0.91	
Triglyceride (mg/dL)			0.228
Baseline	95.9 ± 5.1	126.5 ± 9.8	
Change at 12 week	−7.1 ± 8.0	6.7 ± 7.8	
Total cholesterol (mg/dL)			0.909
Baseline	213.5 ± 4.5	229.0 ± 5.6	
Change at 12 week	−1.3 ± 3.0	−1.8 ± 2.9	
HDL-cholesterol (mg/dL)			0.857
Baseline	58.8 ± 2.3	56.3 ± 1.9	
Change at 12 week	0.22 ± 1.15	0.51 ± 1.1	
LDL-cholesterol (mg/dL)			0.935
Baseline	139.5 ± 4.2	150.2 ± 4.4	
Change at 12 week	−3.2 ± 2.7	−3.5 ± 2.7	
AST (U/L)			0.054
Baseline	24.6 ± 1.4	22.6 ± 1.0	
Change at 12 week	−0.03 ± 0.78	2.2 ± 0.8	
ALT (U/L)			0.054
Baseline	26.2 ± 2.2	23.4 ± 2.1	
Change at 12 week	−0.34 ± 1.17	2.8 ± 1.1	
γ-GTP (U/L)			0.458
Baseline	37.8 ± 4.1	36.0 ± 4.9	
Change at 12 Week	−0.89 ± 1.2	0.40 ± 1.2	

Data are indicated as mean ± S.E. *p* values are calculated by ANCOVA using each baseline value as a covariate.

**Table 3:** Number and ratio of participants who show adverse events that have possible relation to the study design or treatments.

	Test (*n* = 39)	Placebo (*n* = 41)	*p* Value
ALT increased			0.360
Grade 1	1 (3%)	4 (10%)	
AST increased			1.000
Grade 1	0	1 (2%)	
Blood bilirubin increased			0.488
Grade 1	1 (3%)	0	
Blood lactate dehydrogenase increased			0.202
Grade 1	1 (3%)	5 (12%)	
Cholesterol high			0.655
Grade 1	17 (44%)	20 (49%)	
Grade 2	0	1 (2%)	
Creatinine increased			0.353
Grade 1	3 (8%)	1 (2%)	
Hemoglobin increased			1.000
Grade 1	0	1 (2%)	
Hyperglycemia			0.476
Grade 1	5 (13%)	3 (7%)	
Hypertension			0.571
Grade 1	13 (33%)	16 (39%)	
Grade 2	7 (18%)	10 (24%)	
Hypertriglyceridemia			0.080
Grade 1	1 (3%)	6 (15%)	
Grade 2	0	1 (2%)	
Hyperuricemia			0.111
Grade 1	3 (8%)	0	
Serum amylase increased			1.000
Grade 1	0	1 (2%)	
Weight loss			1.000
Grade 1	1 (3%)	2 (5%)	
White blood cell decreased			0.111
Grade 1	3 (8%)	0	

## Data Availability

The data presented in the study are available in article.

## Supplementary Materials

The following are available online at https://www.mdpi.com/article/10.3390/nu14214492/s1, Table S1: Changes in other monitored parameters in this study; Supplementary Data: Preliminary experiment on high-fat diet (HFD)–induced obesity model mice using IJH-SONE68-fermented pineapple juice; Figure S1: The difference in changes in body weight gain observed in HFD-induced-obesity mice with the simultaneous intake of IJH-SONE68-fermented pineapple juice; and Figure S2: The difference in the amount of visceral fat in HFD-induced-obesity mice with the simultaneous intake of IJH-SONE68-fermented pineapple juice.
