# Supplementary material for: Plant-Derived Lactobacillus paracasei IJH-SONE68 Improves the Gut Microbiota Associated with Hepatic Disorders: A Randomized, Double-Blind, and Placebo-Controlled Clinical Trial
Source: Nutrients. 2022 Oct 26;14(21):4492. doi: 10.3390/nu14214492 (PMC9657077; doi:10.3390/nu14214492)
Supplement: Supplementary file 1 [file nutrients-14-04492-s001.zip › Suppl_Data.pdf]

Supplementary Data

Preliminary experiment on high-fat diet (HFD)–induced obesity model mice using IJH-SONE68-fermented pineapple juice

Materials and Methods

LAB strain culture conditions

For seed cultivation, Lactobacilli MRS broth (Becton, Dickinson and Company, Franklin Lakes, NJ, USA) was used as a culture medium for *Lactobacillus paracasei* IJH-SONE68. After cultivation for 2 days at 28°C, the collected bacterial cells were washed with and resuspended in sterile distilled water; then aliquots of the cells were inoculated into the sterile pineapple juice supplemented with 1 (v/v) % sake lees to cultivate at 28°C for 2 days.

Preparation of diet for mice

To establish HFD-induced model mice, a D12492 diet (Research Diets, Inc., New Brunswick, NJ, USA) was used. The diet was crushed and placed in a glass feeder. If necessary, fermented pineapple juice (7 mL) was added to the HFD (120 mL) at a 10-fold serial dilution. As a negative control, mice fed with a regular diet of "MF" (Oriental Yeast Co., Ltd., Tokyo, Japan) were also used.

Study design and ethics statement

Male C57BL/6 Jcl specific pathogen–free (SPF) 7-week-old mice were purchased from CREA Japan, Inc. (Tokyo, Japan). After a 1-week acclimation period with a regular diet,

they were divided into five experimental groups of five mice each and started on an HFD except for the negative control (lean control) group. The mice were housed in plastic cages and kept under the following conditions: 20–26°C, 40–60% humidity, and a 12 h light/12 h dark cycle. All mice could freely access diet and water, which were changed every week, and the amount of daily food intake in each group was monitored. The body weight of each mouse was also recorded each week. To distinguish each mouse in the same cage, each of their tails was painted a different color using felt-tip markers (Animal Marker, Muromachi Kikai, Co., Ltd., Tokyo, Japan). After the 12-week intake period, the mice were euthanized via inhalation of anesthesia with isoflurane, and visceral fat was collected from each one. The changes in body weight from the baseline and the amount of visceral fat were statistically compared using a Tukey–Kramer test. Statistical analyses were performed using SPSS 17.0 software (SPSS Japan, Inc., Tokyo, Japan).

The animal experiment in the present study was conducted in accordance with the Guidelines for the Care and Use of Laboratory Animals of Hiroshima University. All experimental protocols for the present study were approved by the committee of the Research Facilities for Laboratory Animal Science of Hiroshima University (approval number A17-49).

Results

As shown in Figures S1 and S2, the body weight gain and visceral fat accumulation were suppressed by 31.1% and 60.3%, respectively, with statistical significance in the 10-fold-diluted sample-feeding group compared with obesity control group. Although the same parameters observed in the undiluted-sample feeding group were also suppressed, there

were only statistical tendencies against the obesity control. The result in the 100-fold-diluted group indicated that over-dilution is not an effective process.

Figure Legends

Figure S1. The difference in changes in body weight gain observed in HFD-induced-obesity mice with the simultaneous intake of IJH-SONE68-fermented pineapple juice. Values are indicated as the mean ± standard error (S.E.). Statistical analysis was performed using the Tukey–Kramer multiple comparisons test (*$p < 0.05$).

Figure S2. The difference in the amount of visceral fat in HFD-induced-obesity mice with the simultaneous intake of IJH-SONE68-fermented pineapple juice. Values are indicated as the mean ± S.E. Statistical analysis was performed using the Tukey–Kramer multiple comparisons test (*$p < 0.05$).

Figure S1

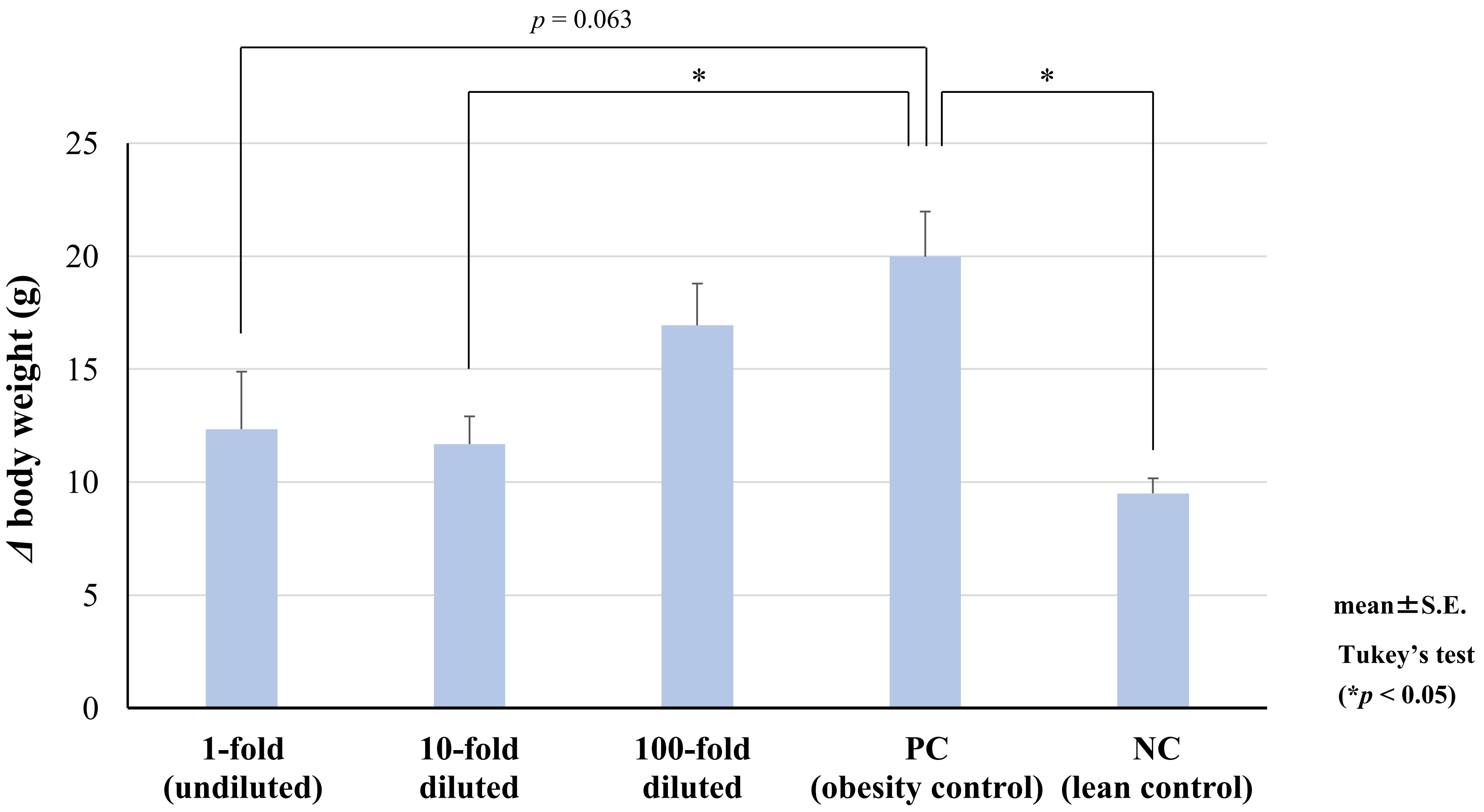

p = 0.063
*
*
Δ body weight (g)
0
5
10
15
20
25
1-fold (undiluted)
10-fold diluted
100-fold diluted
PC (obesity control)
NC (lean control)
mean±S.E.
Tukey's test
(*p < 0.05)

Figure S2

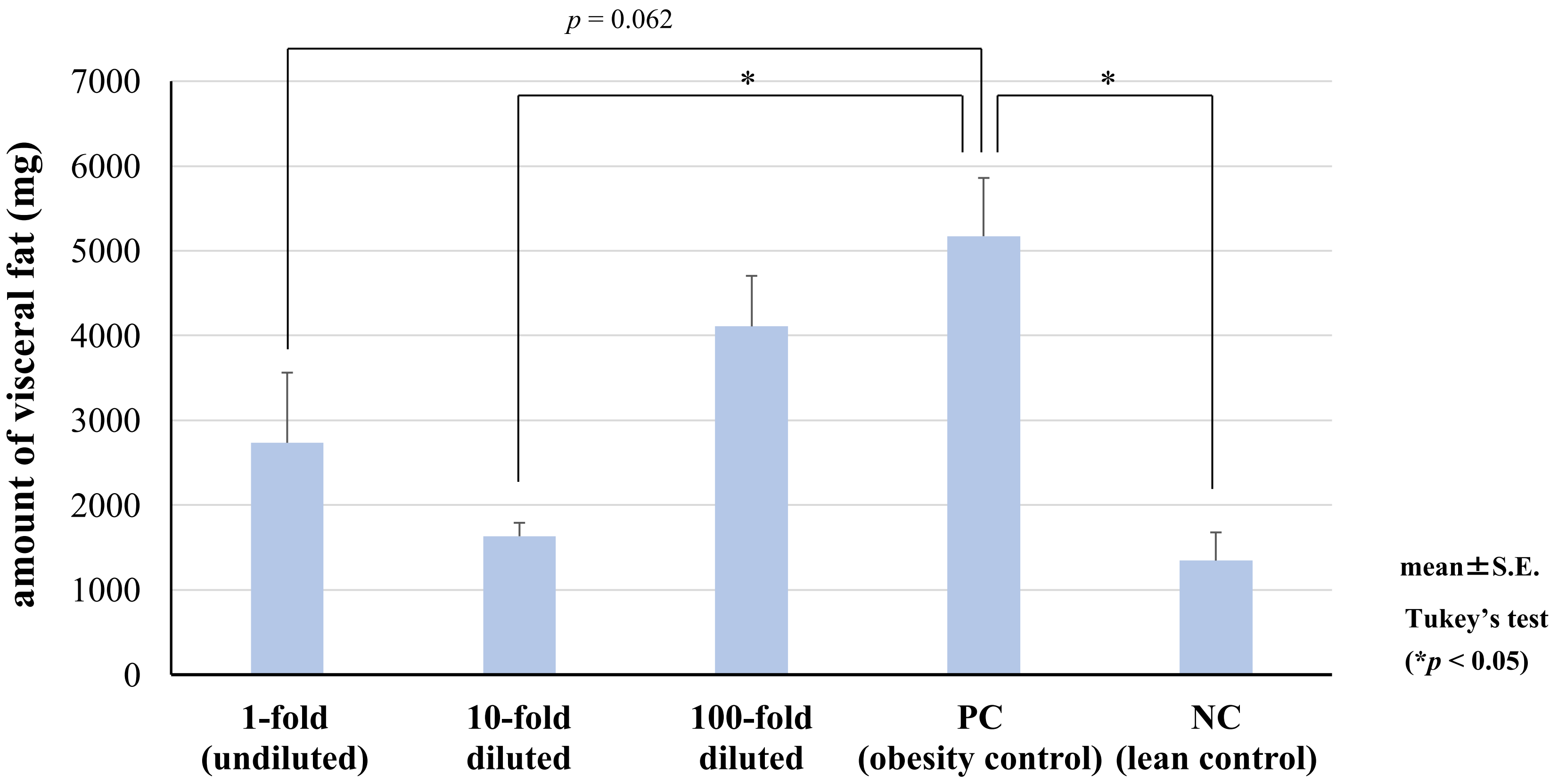

p = 0.062
*
*
amount of visceral fat (mg)
7000
6000
5000
4000
3000
2000
1000
0
1-fold (undiluted)
10-fold diluted
100-fold diluted
PC (obesity control)
NC (lean control)
mean±S.E.
Tukey's test
(*p < 0.05)
