# Supplementary material for: Plant-Derived Lactobacillus paracasei IJH-SONE68 Improves the Gut Microbiota Associated with Hepatic Disorders: A Randomized, Double-Blind, and Placebo-Controlled Clinical Trial
Source: Nutrients. 2022 Oct 26;14(21):4492. doi: 10.3390/nu14214492 (PMC9657077; doi:10.3390/nu14214492)
Supplement: Supplementary file 1 [file nutrients-14-04492-s001.zip › TableS1.pdf]

Table S1. Changes in other monitored parameters in this study.

	Test (n = 39)	Placebo (n = 41)	P value
Body weight (kg)			0.901
Baseline	72.3 ± 1.6	68.9 ± 1.3	
Change at 12 week	-0.23 ± 0.24	-0.19 ± 0.24	
Systolic blood pressure (mmHg)			0.392
Baseline	125.0 ± 2.4	126.0 ± 2.2	
Change at 12 week	-1.7 ± 1.5	0.12 ± 1.47	
Diastolic blood pressure (mmHg)			0.992
Baseline	76.8 ± 2.0	76.3 ± 1.6	
Change at 12 week	0.009 ± 1.037	-0.005 ± 1.015	
Albumin (g/dL)			0.966
Baseline	4.38 ± 0.04	4.40 ± 0.04	
Change at 12 week	-0.051 ± 0.043	-0.048 ± 0.043	
Alkaline phosphatase (U/L)			0.931
Baseline	223.4 ± 9.6	206.8 ± 9.5	
Change at 12 week	-2.2 ± 3.9	-1.8 ± 3.8	
Amylase (U/L)			0.936
Baseline	73.9 ± 2.9	74.3 ± 4.8	
Change at 12 week	1.3 ± 1.5	1.1 ± 1.5	
Total bilirubin (mg/dL)			0.469
Baseline	0.71 ± 0.04	0.63 ± 0.03	
Change at 12 week	0.002 ± 0.032	-0.031 ± 0.033	
Blood urea nitrogen (mg/dL)			0.403
Baseline	14.5 ± 0.5	14.0 ± 0.5	
Change at 12 week	-0.14 ± 0.37	-0.58 ± 0.37	
Cholinesterase (U/L)			0.380
Baseline	358.2 ± 8.6	363.2 ± 11.1	
Change at 12 week	-3.4 ± 4.5	2.3 ± 4.5	
Creatinine (mg/dL)			0.317
Baseline	0.78 ± 0.02	0.72 ± 0.02	
Change at 12 week	-0.004 ± 0.011	-0.020 ± 0.011	
Lactate dehydrogenase (U/L)			0.044
Baseline	204.7 ± 5.2	196.7 ± 4.9	
Change at 12 week	-1.9 ± 4.5	10.8 ± 4.4	
Total protein (g/dL)			0.245
Baseline	7.34 ± 0.06	7.50 ± 0.06	
Change at 12 week	-0.056 ± 0.055	0.036 ± 0.056	
Ureic acid (mg/dL)			0.428
Baseline	6.1 ± 0.2	5.6 ± 0.2	
Change at 12 week	0.008 ± 0.112	-0.12 ± 0.11	

Data are indicated as mean ± S.E.

P values are calculated by ANCOVA using each baseline value as a covariate.
